# Resilience anchors for children in an out-of-home care institution during and after COVID-19

**DOI:** 10.3389/fpsyg.2023.1189739

**Published:** 2023-10-02

**Authors:** Macalane Junel Malindi, Johnnie Hay

**Affiliations:** School of Psychosocial Education, North-West University, Potchefstroom, South Africa

**Keywords:** COVID-19, multisystemic interventions, out-of-home care, resilience, resilience resources, resilience risks, social ecology of resilience theory

## Abstract

Growing numbers of children of all ages grow up in out-of-home care institutions due to personal and socioecological risk variables that destabilized their families of origin. In the aftermath of the COVID-19 pandemic which disrupted lives and development, there is particular interest in how children who grow up in out-of-home care institutions cope and develop. This paper reports the findings of a study that sought to document anchors of resilience in children who resided in a care institution run by a non-governmental, church-based welfare organization in one of the central provinces of South Africa. In line with recent developments in childcare, the organization mainly functions *via* smaller child and youth group homes across the province (compared to bigger children’s homes in the past). In our qualitative, phenomenological study, we used the participatory, child-friendly, and less intrusive draw-and-write technique to generate data. We asked the 20 participating children of one of these group homes to make drawings that mirror their lives, and to write paragraphs in which they described their drawings. All were school-going children in care, aged from 12 to 19. There were 11 girls and 9 boys in the study, and one of these identified as “other.” The grades ranged from 7 to 12 and they spoke African languages, namely Afrikaans, Sesotho, Setswana and IsiXhosa. We used inductive content analysis to process the data, and the findings indicate that, notwithstanding personal and socioecological risks during and after the COVID-19 pandemic, the resilience of the participants was anchored by a number of universal personal strengths as well as socioecological resources.

## Introduction

Considerable numbers of at-risk children who hail from families where risks abound grow up in residential, out-of-home care institutions worldwide (Mayer, 2019, p. 550; UNICEF, 2021). The reasons for this phenomenon range from personal to socioecological risks that impair holistic development in children. However, it should be noted that sometimes, complex combinations of these personal and socioecological risks account for poor developmental outcomes in children at risk. Well-known examples of these risks are traumatic occurrences such as neglect, and physical, sexual, and psychological abuse, often by the hands of parents or legally responsible caregivers (Leve et al., 2012, p. 1,197; Vandervort et al., 2012, p. 1). Extremes of poverty and family instability account for a significant percentage of children in out-of-home care contexts (Malindi and Molahlehi, 2020, p. 281). Furthermore, overwhelming natural occurrences such as pandemics, earthquakes, floods and manmade disasters such as veldfires and wars disrupt families and child development, causing especially orphans, to subsist in out-of-home care institutions or temporarily established camps.

It is accepted that children thrive and cope resiliently in contexts where there is stability, an established routine, certainty, access to adequate housing, nutrition, medical care, and secure relationships with adults, as well as quality learning opportunities (Sandstrom and Huerta, 2013, p. 4). Any significant instability in the early years may negatively impact child development and the capacity to cope adaptively in the context of such risk. The recent COVID-19 pandemic, which was an overwhelming natural disaster, created much instability, fear, anxiety and uncertainty in the lives of young people worldwide (Ganie and Mukhter, 2020, p. 80), including those in care institutions. Therefore, the question that interested us as authors was how children in care settings cope(d) adaptively during and after the pandemic.

It is important to note that for some time, there has been renewed interest in the resilience of children who have experienced natural and other manmade disasters (Masten, 2014, p. 6). In this regard the capacity of young people at risk to cope adaptively in the context of the COVID-19 pandemic became a node of interest. Researchers and mental health practitioners have become interested in how young people navigated their pathways through natural disasters. Perspectives on the constellation of protective resources and services that anchored the resilience of these young people who were at risk of poor developmental outcomes, were and are explored.

A number of studies have examined adaptive coping in children in their families of origin who were affected by the COVID-19 pandemic. In the context of the COVID-19 pandemic it was discovered that children exhibited fear, experienced problems with online learning, a deterioration in physical and mental health, reduced physical activity, and demonstrated an increased interest in gadgets and body weight (Shmatova and Razvarina, 2022, p. 140). Other researchers noted that children demonstrated negative affect and behavior, and experienced episodes of neglect or abuse, isolation, boredom, lack of free outdoor play and poverty (Ganie and Mukhter, 2020, p. 80).

Children in care institutions were perhaps in more difficult circumstances, since the abrupt changes brought about by the pandemic added to the changes they had already experienced in their lives. As Treier et al. (2022, p. 951) noted, at-risk children with affective dysregulation and those in out-of-home care contexts exhibited elevated reactivity to stressors and maladaptive emotional regulation coping strategies. This is indicative of vulnerability. It is accepted that early life experiences tend to shape development and learning in children (Sandstrom and Huerta, 2013, p. 4). It should be borne in mind that children in care institutions have histories of early life trauma and instability. To this the COVID-19 pandemic added a layer of risk in those children who were already in a vulnerable position in care institutions.

While it is accepted that the COVID-19 pandemic negatively influenced psychological development in some learners, others demonstrated the capacity to remain resilient in the context of risk and adversity posed by the pandemic (Panzeri et al., 2021, p. 17). In South Africa, it would be important for researchers to purposefully examine how the COVID-19 pandemic impacted development and resilience in children in out-of-home care institutions and document the resilience anchors that enable(d) them to cope resiliently. This served as motivation for this study.

### The resilience phenomenon in youth in out-of-home care settings—internationally and in South Africa

Resilience researchers acknowledge that the resilience phenomenon is characterized by ambiguity and not easy to define since it is complex and influenced by the context (Dass-Brailsford, 2005, p. 575; Ungar, 2011, p. 1). However, there is consensus that resilience depends strongly on personal and socioecological factors inside and around the youth at risk (Ungar, 2005, p. 429; Ungar, 2011, p. 1). In other words, there is consensus that positive adjustment in youth at risk depends on reciprocal, dynamic, contextually influenced bi-directional interactions between them and their social and physical ecologies (Theron and Donald, 2013, p. 61). Young people at risk depend on personal strengths and active support systems such as families, schools, and peers to cope adaptively in the context of risk and adversity (Theron and Donald, 2013, p. 58). Young people should have access to multiple resources from multiple systems to cope adaptively (Theron et al., 2022, p. 7). In other words, young people develop resilience in the context of risk if they receive support from schools, families and their communities (Ungar et al., 2019, p. 616–617). Contextual realities therefore mold the capacity to cope resiliently in youth at risk in depressed contexts (Theron, 2015, p. 635–636). However, for young people in youth care settings, support systems are often not adequate.

They typically have histories of traumatic experiences, are homeless and hail from unstable homes where resources such as food are compromised. Care institutions are seen as an alternative, safe havens for vulnerable youth since they cater for the basic needs of orphans and homeless, at-risk children, who typically have histories of physical, sexual, emotional, and psychological abuse and neglect (Leve et al., 2012, p. 1,197). However, there are differing views as to whether out-of-home care institutions serve as what Theron and Engelbrecht (2012, p. 265) call *microsystemic strongholds* for children who are at risk of poor developmental outcomes.

Some researchers express a rather pessimistic view of care institutions’ potential to serve as safe havens for at risk children and youth to enable adaptive coping. Taylor et al. (2018, p. 83) argue that although children growing up in residential care institutions cope resiliently because of personal strengths such as future focus and motivation and interpersonal relationships, they are still more vulnerable compared to their at-risk peers who grow up in their own homes.

In a South African study, Hlungwani and Van Breda (2022, p. 147) noted that care institutions are typically restrictive by nature, and thus fail to fully prepare young people for life post-care. They argue that some of the children who exit care at maturity often struggle to cope adaptively in the wider, more open society. However, according to Farmer et al. (2021, p. 1), many young people who exit care demonstrate resilience despite the risk and adversity they had previously experienced. A view is also expressed that some of the children in care institutions are at risk of maltreatment, often with deleterious mental health consequences (Gusler et al., 2020, p. 455). Mayer (2019, p. 550) emphasizes that exposure to trauma, acute or chronic, can be damaging to young children’s development.

According to Huffhines et al. (2020, p 439) children in care institutions who experience maltreatment exhibit internalizing and externalizing problems and experience more psychiatric hospitalizations. Despite this, some researchers noted that children in care institutions demonstrated the capacity to remain resilient in the context of maltreatment, notwithstanding the risks and shortcomings referred to above.

In a study that examined thought problems and aggression among children in out-of-home care, Farley et al. (2022, p. 795) found that placement in care institutions provided a structure that reduced the incidence of aggression in children. This strengthens the view that children require structure and a measure of predictability in their lives to develop normatively (Varelas et al., 2015, p. 517). Another study found that when youth in care institutions had positive relationships with caseworkers, they attained positive emotional, behavioral and cognitive school engagement outcomes (Jaramillo and Kothari, 2022, p. 399).

School engagement was found to be potent in promoting resilience in children with street life experiences in a care institution in South Africa (Malindi and Machenjedze, 2012, p. 77; Theron et al., 2022, p. 1). School engagement extends opportunities to children in care to build social competencies, participate in learning activities and develop meaningful social connections with peers and teachers that enable resilience (Kothari et al., 2020, p. 914).

Thomas et al. (2022, p. 61) noted that resilience among children in an out-of-home care institution in Kerala, India was anchored in positive relationships among the children at the shelter. Such relationships foster a sense of belonging, an important factor of resilience. Another study in the UK confirmed the importance of a resilience-promoting environment and emotionally supportive networks in promoting resilience among children in out-of-home care institutions (Furey and Harries-Evans 2021, p. 404).

Youth who are weaned from institutional care and transition from dependence to independence require adequate mentoring to be able to cope resiliently in the wider outside world (Sulimani-Aidan et al., 2019, p. 345). A South African study that looked at the resilience of girls leaving a care institution showed that they remained resilient because they readily embraced motherhood (and related responsibilities), faith and an attitude of gratitude for the care they received in the care institution (Hlungwani and van Breda 2020, p. 918). In this regard, Gusler et al. (2020, p. 455) advise that it would be important for researchers to examine the processes that enable some of the children in care institutions to remain resilient despite maltreatment. This will allow for context-specific interventions to be developed to ameliorate the plight of children at risk of poor developmental outcomes.

### Our theoretical perspective on resilience

We view youth resilience through the lens of Social Ecology of Resilience Theory (SERT) of Ungar (2011). This theory consists of four basic principles, namely decentrality, complexity, atypicality and cultural relativity (Ungar, 2011, p. 4). In line with the principle of decentrality, young people should be decentered when we seek evidence of resilience (Ungar, 2011, p. 4). This implies that the role of social ecologies, consisting of families, schools and communities in determining adaptive coping in youth at risk should be recognized (Ungar, 2012, p. 17). With regard to the principle of complexity, Ungar argues that risks, resilience resources and resilience are complex processes that deter us from confidently predicting children’s developmental trajectories. Individual attributes or strengths potentiate resilience; however, these attributes combine in complex ways with resources sourced from social and physical ecologies to enable adaptive coping in young people at risk (Panter-Brick and Eggerman, 2012, p. 369; Ungar, 2012, p. 17).

The SERT’s third principle is atypicality. Young people sometimes use atypical ways to cope in the context of risk (Ungar, 2011, p. 7–8). This is reminiscent of hidden resilience, noted in young people with street life experiences in a South African care institution (Malindi and Theron, 2010, p. 324). These young people vandalized payphones to obtain money and teased one another—aligned with hidden resilience where resilient behaviors are not always viewed as constructive.

Through the principle of cultural relativity, SERT demonstrates how culture can enable or compromise resilient coping in young people at risk (Panter-Brick and Eggerman, 2012, p. 369; Sugawara et al., 2021, p. 2; Ungar, 2011, p. 8). This implies that resilience is nuanced by culture.

Based on our focus of interest, a discussion of the phenomenon of resilience and the theoretical perspective of SERT, our purpose and research question were refined.

### The purpose and research question of the study

Our purpose was to determine which resilience anchors helped youth in an out-of-home care setting to cope adaptively when confronted with the COVID-19 pandemic.

Our research question therefore was: *Which resilience anchors did youth in a South African care setting utilize to cope adaptively while facing the COVID-19 pandemic and its aftermath?*

## Methodology

There has been a call that young people be studied directly through methodologies that take their levels of development into account (Driessnack, 2005, p. 415; Malindi and Molahlehi, 2020, p. 299). In this study, we heeded that call and selected the approach that would be suitable for the participants in our study. We adopted a qualitative research approach and used the draw-and-write technique to generate data. The feasibility of this technique to generate data with traumatized children was established with orphans in a care institution (Machenjedze et al., 2019, p. 72).

Our study involved 20 children in a group home care institution who volunteered to take part in the research. Consent was obtained from the non-governmental organization as well as the house parents of the group home. Before visiting the setting, we sent a detailed research letter as well as assent forms to the parents. Assent from the volunteer participants was obtained by the house parents. There were 11 girls and 9 boys in the sample, one of whom identified as “other.” The participants spoke the dominant African languages in the area, namely Afrikaans (*N* = 13), Sesotho (*N* = 3), Setswana (*N* = 2), and IsiXhosa (*N* = 2). All the participants confessed to being Christians, were in school and in grades ranging from 7 to 12 and they were aged from 12 to 19. We did not ask for confidential demographic data such as personal and family history—in line with the South African Protection of Personal Information Act 4 of 2013. This data would have been helpful but was not the primary focus of the study.

As primary researcher I presented the drawing brief, and pens to the participants. According to the instructions on the brief, they had to make a drawing of what enabled them to cope with their lives during and after COVID-19—and to write a paragraph in which they described their drawings. They were allowed to write their narratives in any language of their choice. I met the participants in the afternoon after they had returned from school. The house parents shared the contents of the research letter with the participants prior to my arrival—and therefore they had a good idea of what they would be expected to do. I, however, read the letter and instructions once again to ensure certainty, and to respond to any uncertainties they may have had. I asked the participants to give themselves nicknames, which they wrote on the drawing brief, to protect their privacy. The drawing and writing session took approximately 40 minutes. I received the drawing briefs back and provided each participant with a snack pack to thank them for their participation.

Subsequent to the field visit, we processed the data through content analysis. This was done through studying the drawings and reading the descriptions of the drawings several times to gain an optimal understanding of these. Unfortunately, we could not verify our interpretations with the participants, but the secondary researcher served as relatively independent verifier based on his absence during the field work—with the advantage of decoding the data without prejudice. Below, we present the drawings of a better quality and the narratives that describe them. The narratives were not language edited and those that were written in Afrikaans have been translated. Participants with other African home languages wrote their narratives in English.

Some remarks about our positionality as researchers are appropriate at this stage. Both of us work in the School of Psychosocial Education of the Faculty of Education at the North-West University in South Africa and have come a long way together as colleagues. Both of us spent some time in the province where the research was done. The primary researcher speaks isiZulu with a keen interest in resilience and the secondary researcher speaks Afrikaans with a keen interest in education support services within inclusive education. Both of us prefer qualitative investigations where in-depth and rich narratives of participants can be extracted through an interpretivist lens. Both of us operate in the broader fields of educational psychology and learner support—with a belief that the experiences and words of participants should be heard clearly.

### Findings

**Figure 1:**
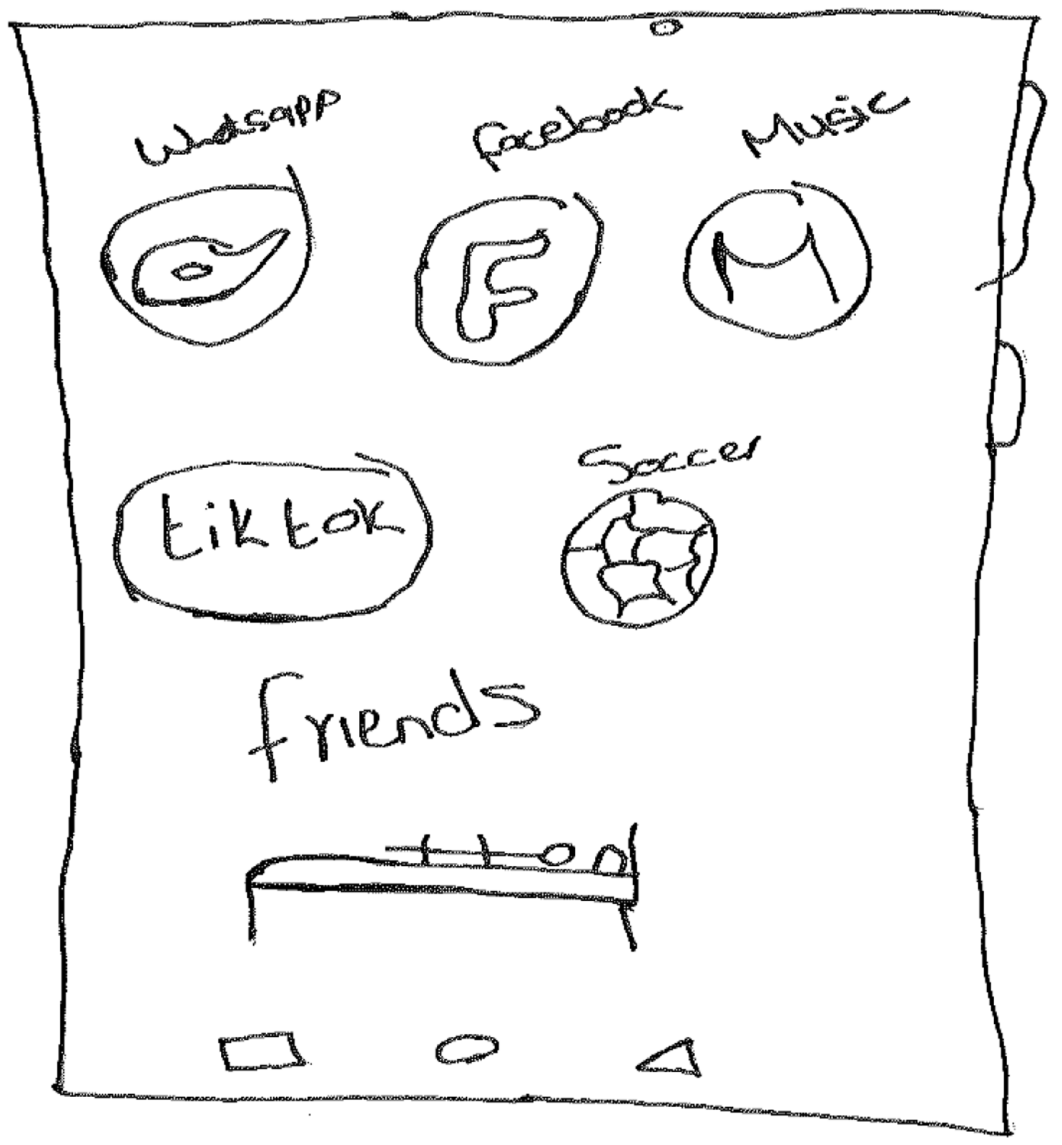
Drawing by Nola.

A general finding that came to the fore is that the participants remained resilient in the context of risk through a constellation of personal and socioecological resilience resources. In this regard, Nola, a 19-year-old participant in Grade 11, who identified as “other,” made a drawing of a mobile phone. The participant wrote the following words on the drawing: *WhatsApp, Facebook, music, TikTok, Soccer, and friends*. The participant then added a human figure lying on a bed (Figure 1).

The participant thereafter wrote the following narrative:

*I drew a cell phone because social media helps me to be strong, during the times where I feel alone. I often read motivational quotes, I write songs and love singing when I feel weak or sad, music helps acts as an escape from reality, my family, and the pain they went through which often leads me to Facebook to search for them when I cannot reach them. Social media helps me to communicate with my friend whom I can trust and talk to about what hurts me, sleeping is also my escape from reality I often sleep when I do not feel alright, playing soccer with my friends helps to ease my mind and lastly my girlfriend also helps me quite a lot when I am at my lowest.*

The narrative shows that on a personal level, Nola coped adaptively through *social media* when he felt alone. It is important to note that the participant tried to connect to his family through Facebook. Social media enabled the participant to communicate with peers about painful moments and thus benefit from social capital. Additionally, on a socioecological level, his *girlfriend provided social support* when it was needed. Earlier studies demonstrated that, in the context of disasters, social media was used effectively to enable social support and a sense of belonging (Dufty, 2012, p. 40; Jurgens and Helsloot, 2017, p. 79; Mano, 2020, p. 460; Senekal et al., 2022, p. 10–11). The participant *read motivational quotations* and used his talent for *writing songs and singing* when he felt weak or sad. It is noteworthy that the participant escaped from their painful reality through *sleeping* and *music*. Another study found that sleep enables one to recover from, adapt or resist a stressful event (Guida et al., 2023, p. 3). While a study by Kegelaers et al. (2021, p. 1,279) shows that professional musicians and music students exhibited mental health issues such as depression and anxiety, a study by Rosenberg et al. (2021, p. 6) shows that listening to music enabled posttraumatic resilience.

**Figure 2:**
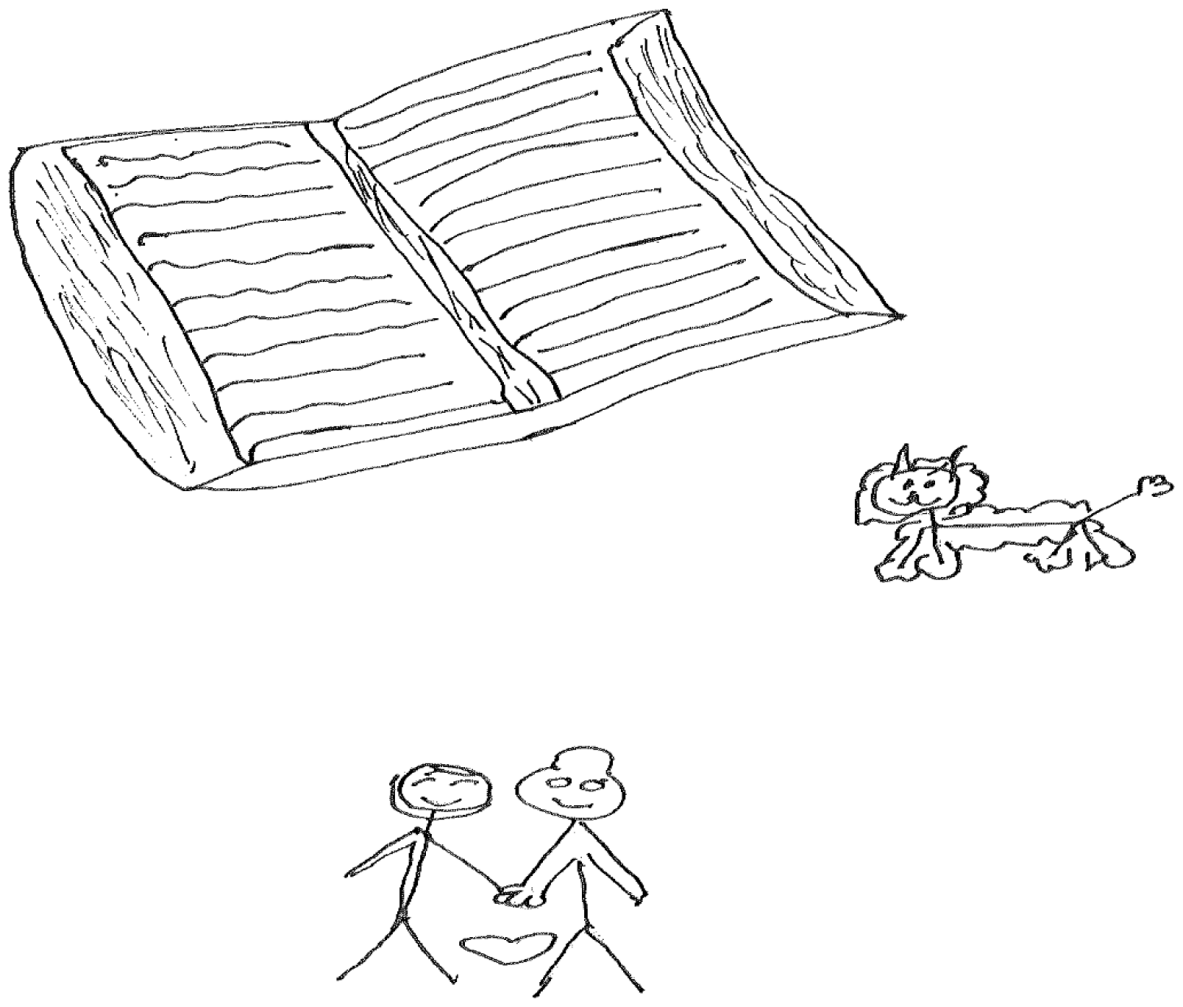
Drawing by Lexy.

Lexy, a 15-year-old girl in Grade 9 made a drawing with an open book, a dog and two human figures holding hands. Between the two human figures is a drawing of a heart (Figure 2).

Lexy wrote a narrative in which she described her drawing:

*As ek boeke lees maak dit my baie gelukkig, want ek leer nuwe lese uit die stories*. [When I read books, I feel good, because I learn new lessons from the stories]. *Ek is lief vir mense en diere; dit maak my ook gelukkig*. [I love people and animals; this also makes me happy].

The narrative shows that, on a personal level, Lexy coped resiliently because of the stories she read in books and the lessons she gained from there. Tovey (2021, p. 8) argues that when children are encouraged to read stories, they stand a better chance of understanding themselves and personal and ecological resources needed to overcome adversity. Her *love for people and animals* made her feel happy too, and this probably fed her resilience. Thompson et al. (2014, p. 215) found that animals were important in enabling resilience among vulnerable people in the context of disaster. However, Applebaum et al. (2021, p. 2) found that human-animal bonds can sometimes complicate the experience of adversity in vulnerable groups.

Kaylina, a 15-year-old girl in Grade 10 made a drawing of a book and labeled it, “*Leer*” [learning], a book labeled, “*Bybel*” [Bible], a human figure standing on its own and a group of numerous female human figures, labeled as “*familie*” or [family]. Kaylina wrote the following statement to describe her drawing:

*Om te leer en nie aan die slegte goed te kyk nie en om in beter verhoudings te wees*./[Learning and not focusing on negative events and to be in better relationships].

The above statement shows that on a personal level, the participant coped by *concentrating on studying* and *trying not to focus on negative events* in her life. Modi and Singh (2021)noted the positive thinking enabled resilience in the context of adversity. She also benefitted from having *positive social relationships* on a socioecological level. Although social support has been found to be protective in youth at risk, it was found to be protective in youth experiencing violence in a South African study by Sui et al. (2022, p. 11).

**Figure 3:**
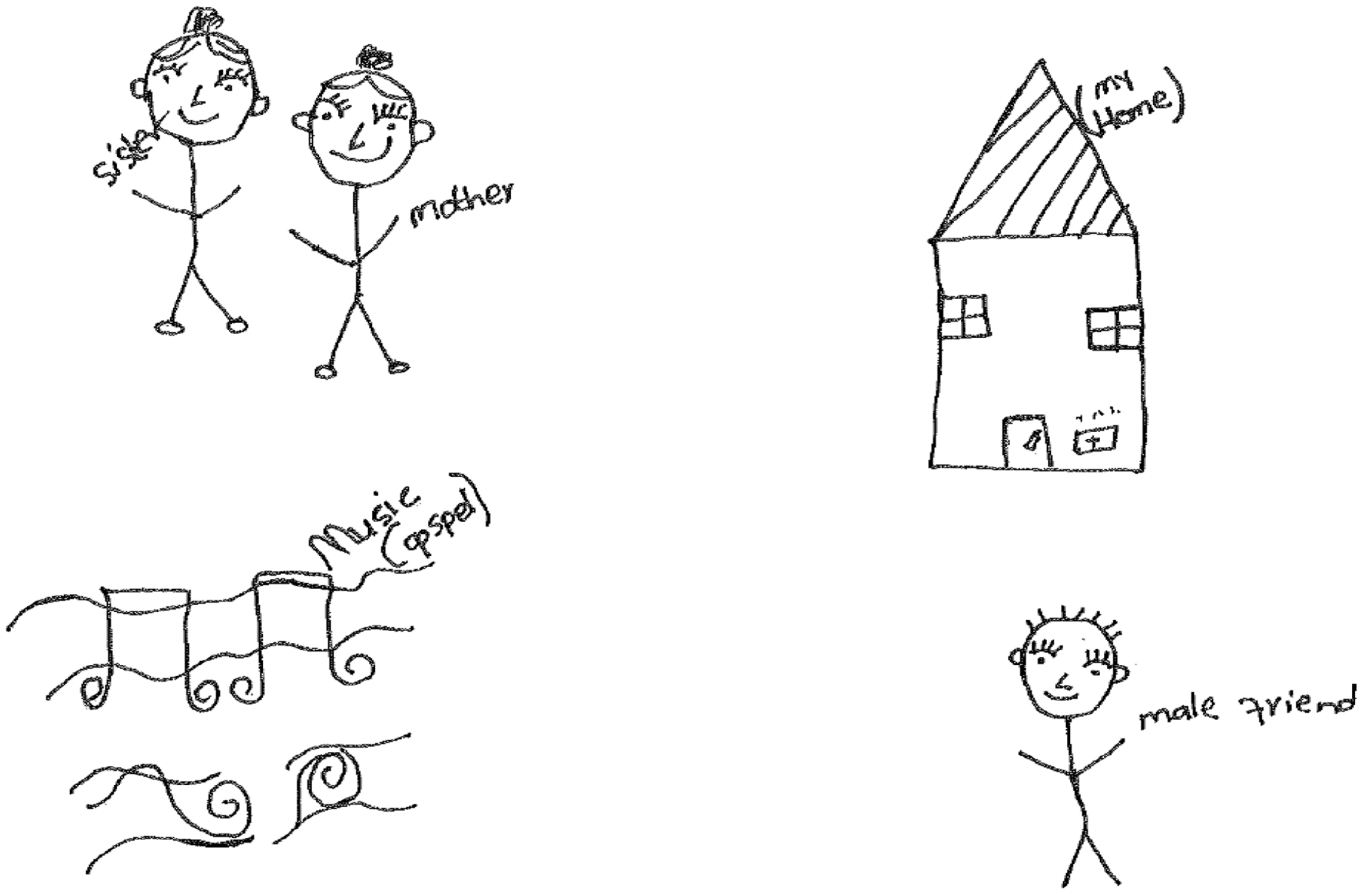
Drawing by Kay-Kay.

Another participant, Kay-Kay, a 17-year-old girl in grade 11 made a drawing showing two human figures labelled *sister and mother*, a house labelled “*my home*”, musical notes, and a male human figure, labelled “*male friend*”. (Figure 3).

Kay-Kay wrote the following paragraph to describe her drawing:

*I feel strong when I am with my sister and mother, they are the source of my strength because when I am sad they give me advice and help me cope through the difficult times, they always make me understand that everything in life happens in life for a reason. Music is also one of the things that make me feel strong because I mostly listen to gospel, I find healing, in some songs I get messages that make me feel better. My male friend is always there for and advice and makes me feel better, my house is also where I get my power from, the reason being is this is where I get space to read my bible and ask God to get me through.*

On a personal level, the participant *listened to gospel music* to feel better, and appreciated the fact that she had a safe place where she could *read her Bible and pray*. Therefore, *having faith* enabled her to cope resiliently on a personal level. A similar finding was made in a study by Ilyashenko et al. (2021, p. 5). In this regard, Ilashenko et al. (2021, p. 5) found that religiosity, religious beliefs and prayer enabled people to calm down in the context of COVID-19. In another study, Gunnestad and Thwala (2011, p. 169) found that children in southern Africa (Zambia and Eswatini) spontaneously mentioned having a religion as a protective factor that enabled their resilience. However, they noted that religiosity rendered some vulnerable to poor developmental outcomes in crisis situations. On a socioecological level, the paragraph shows that the participant coped with difficulties since her *mother and sister were her source of strength*. They made her understand that things happen for a reason. Furthermore, she received *support from her male friend*.

**Figure 4:**
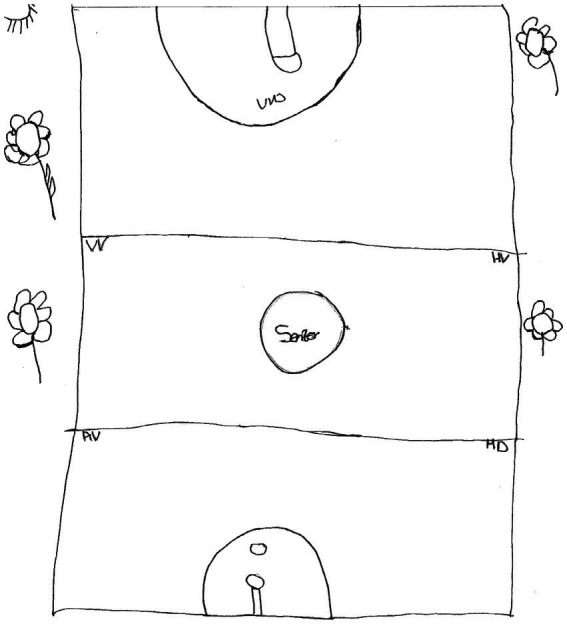
Drawing by Amogelang.

Amogelang, an 18-year-old girl in grade 12, made a drawing of a netball court, decorated with flowers (Figure 4).

Amogelang then wrote the following paragraph:

*Ek het ‘n netbal baan geteken* [I have drawn a netball court]. *Ek wil graag eendag n juffrou wees om kinders netbal te leer* [I would like to become a teacher one day, so that I can teach children netball]. *Dit it sal my ook gelukkig maak as hulle ook eendag groot is en ander kinders netbal leer* [I shall be glad if they could grow up and teach other children netball]. *As die kinders nie wil leer nie, sal ek hulle nie forseer nie, om hulle lief te hê, en hulle nie seermaak nie* [If the children do not wish to learn, I shall not force them, so that I do not hurt them].

The paragraph shows that on a personal level, Amogelang coped resiliently due to personal strengths such as *optimism* and *future focus*. She was optimistic that she would become a teacher in future. This served as motivation to her. Optimism has been found to mediate the impact of COVID-19 related stress on loss of hope (Sorrenti et al. 2022, p. 5,496).

**Figure 5:**
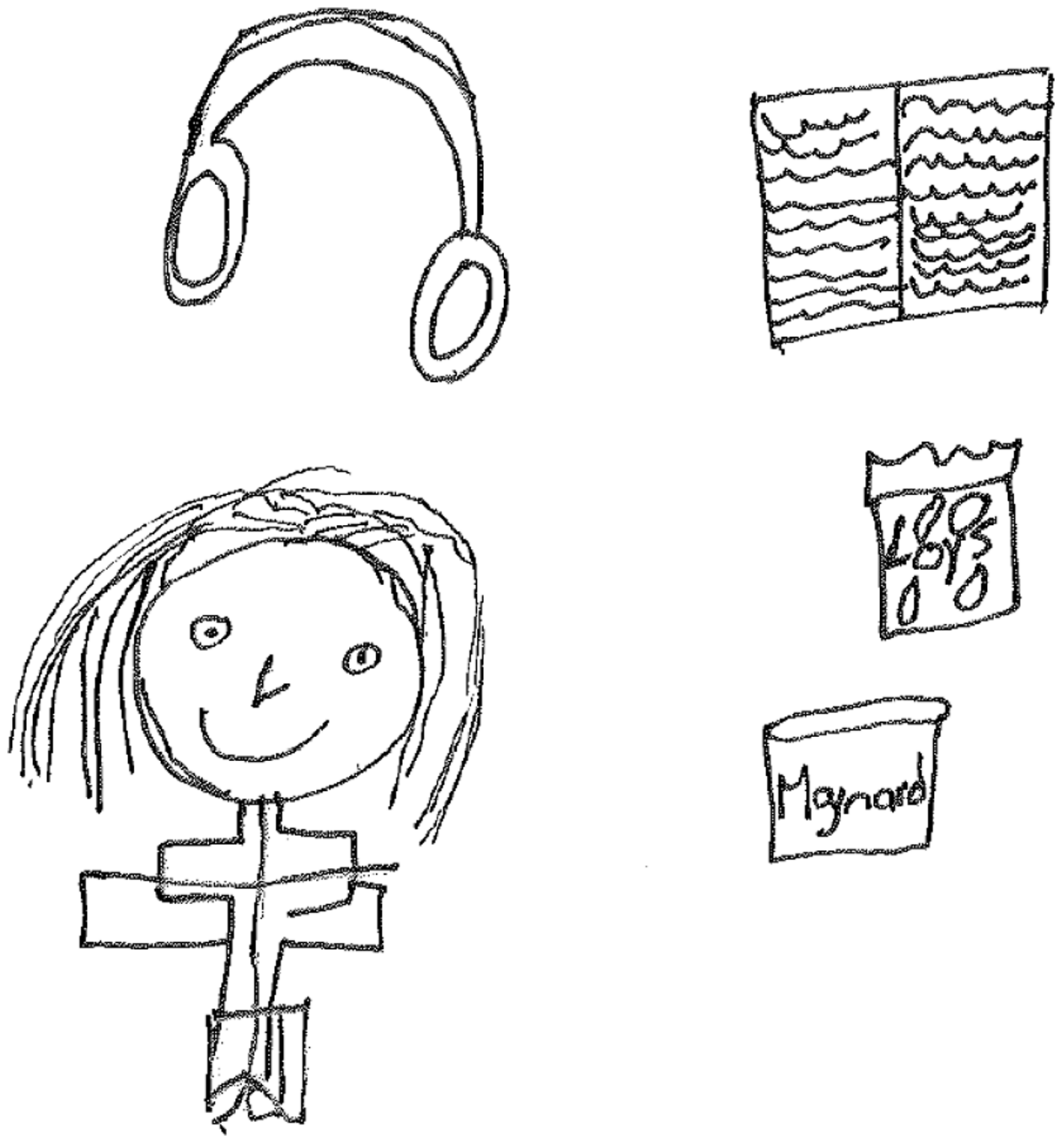
Drawing by Gabriella.

Gabriella, a 17-year-old girl in grade 11 made a drawing of a human figure with headphones above her head. She added the drawing of a book and sweets (Figure 5).

Gabriella said in her narrative:

*Firstly, I drew sweets because when I feel stressed I tend to eat a lot of sweets, I drew earphones because I listen to music when I am sad and angry, it helps to calm me down. The person I drew represents my family, they are the reason why I am fighting. I have so much hope and I love them so much because every day when I wake up I do it for them because they need me and I do it because I want to achieve my goal and to make my mother proud, the book is for the Bible because when I pray and read the bible I feel that I have a lot of strength and everything, but I do it for them, When they look at me they should feel a sense of strength and I want to change my home situation, thanks for inviting me.*

The paragraph above shows that on a personal level, Gabriella remained resilient because of personal strengths such as *comfort eating* of sweets, *listening to music* to calm down, *having hope*, *reading the Bible, and praying*. On the socioecological level, Gabriella benefitted from meaningful *connections to her family* and the *desire to succeed* to make them proud. It is important to note that comfort eating has been found to be one of the outcomes of emotional stress related to COVID-19 (Salazar-Fernández et al., 2021, p. 1).

**Figure 6:**
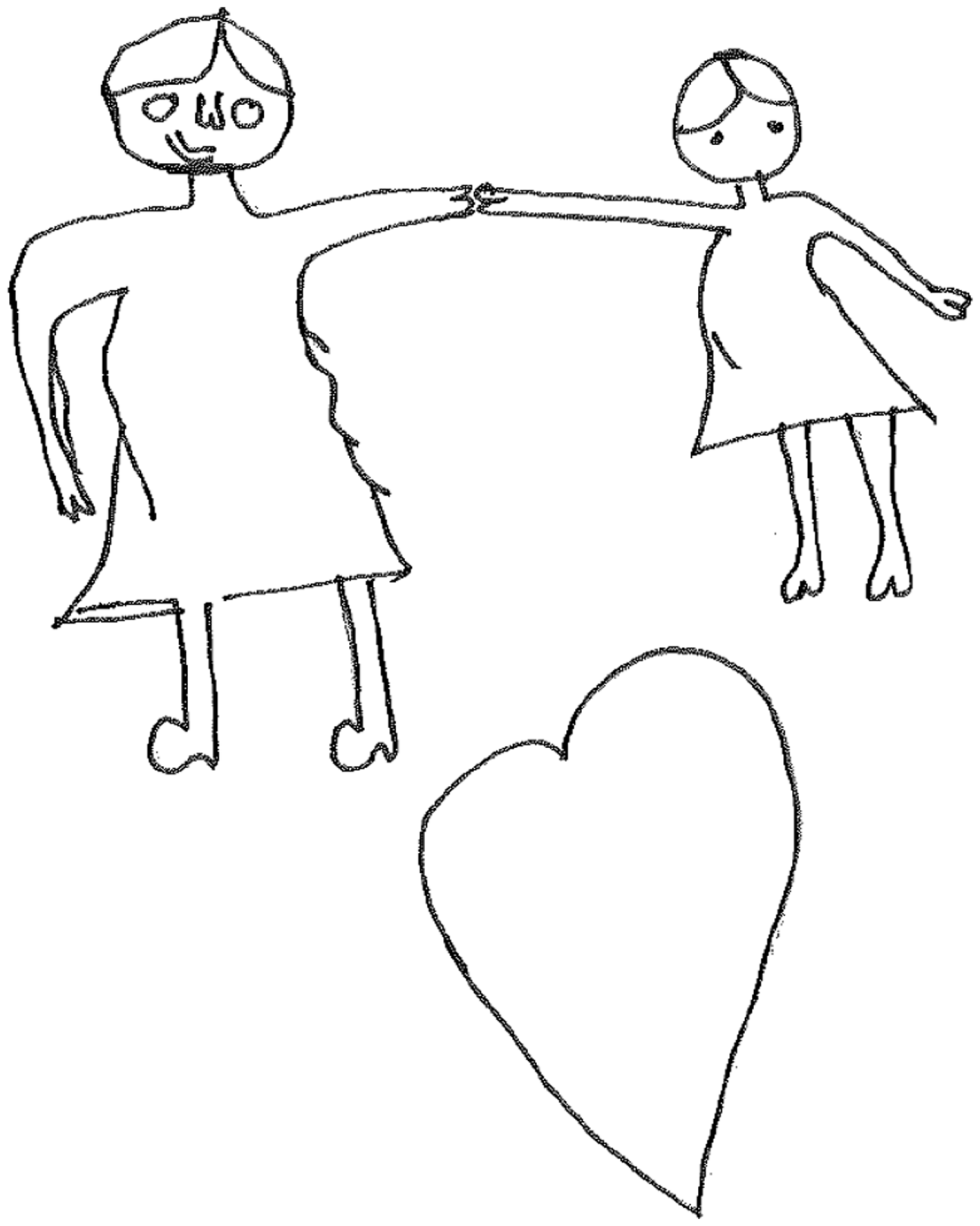
Drawing by Beauty.

Beauty, a 12-year-old girl in grade 7 made a drawing of two human figures holding hands. In between them, she made a drawing of a heart (Figure 6).

Beauty wrote the following paragraph:

*My family helps me to be strong at any time and help me to cope or the caretaker gives me some more advice, I’ve drawn my family and my caretaker, and I’d like to thank Mr. Malindi [one of the researchers] for helping me with this situation and I’d also like to thank Mme XXX [name withheld] for giving me advise.*

On a personal level, the paragraph shows that Beauty had an *attitude of gratefulness*, that has been found to enhance resilience. Gratitude before the pandemic promoted well-being in the midst of the COVID-19 pandemic (Kumar et al., 2022). The above paragraph shows that on a socioecological level, Beauty coped adaptively due to her *family*, and the *caretaker who served as an active support system*. The family made her strong and helped her cope while the caretaker, whose name is withheld gave her useful advice.

**Figure 7:**
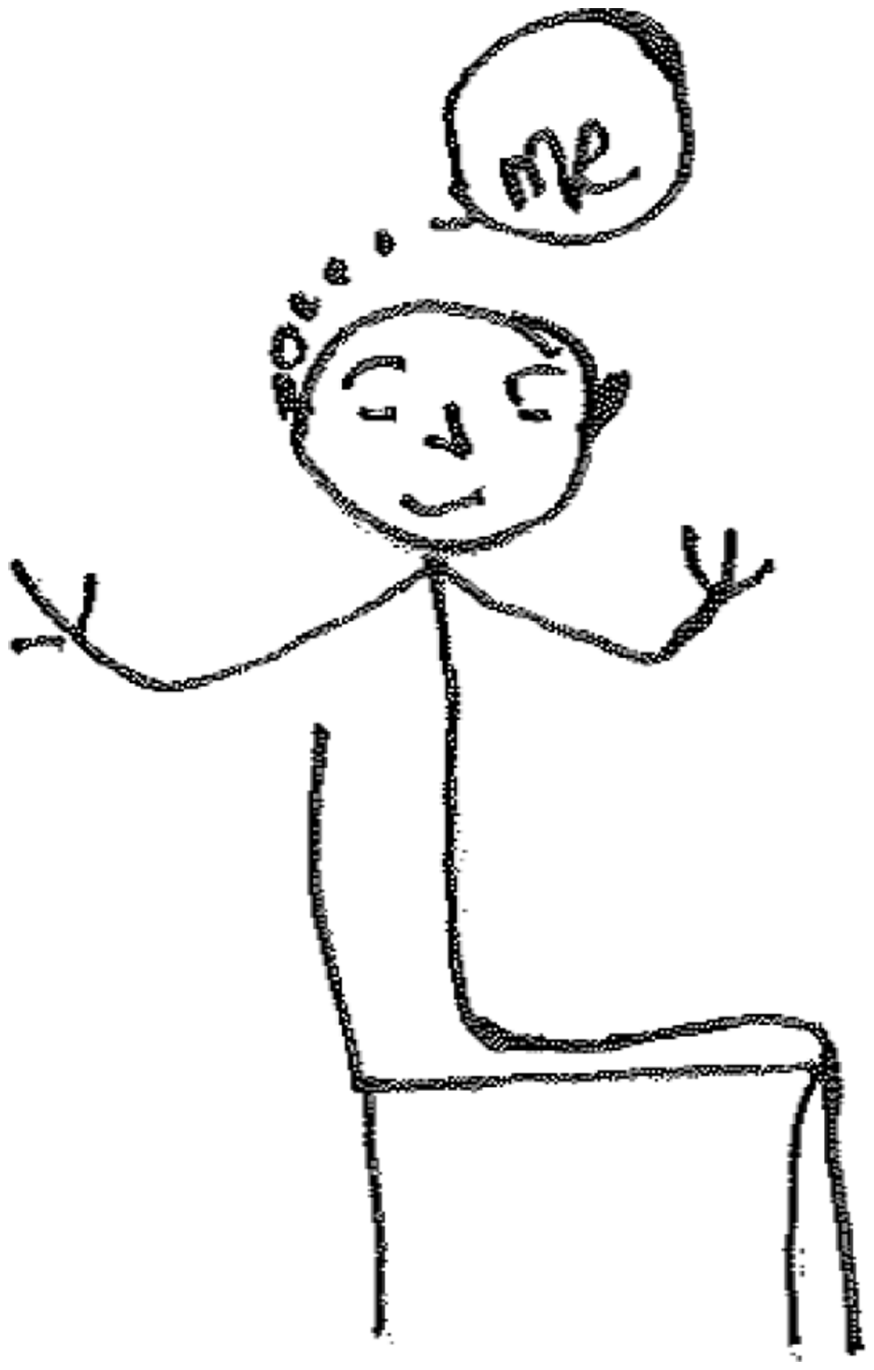
Drawing by Paulinah.

Paulinah, a 17-year-old girl in grade 10 made her drawing that shows a human figure sitting on a chair with arms outstretched. There is a circle above the human figure with the word, “me” inside (Figure 7).

Paulinah wrote the following paragraph:

*I drew myself, when I am imagining in class, sometimes I like to feel lonely but some other times I feel like it’s a good thing for me to be alone. It gives people the peace that they need and the important thing is that it helps me a lot to be alone and think*.

The paragraph shows that on a personal level, Paulinah might be *at risk and psychosocially vulnerable*, as she is intentionally withdrawing socially from peers and teachers. However, she might be an introverted person, which is a personality orientation and not necessarily a risk factor.

Another participant, namely Martha, a 14-year-old girl in grade 7 made a drawing of a male and a female human figure, labeled “*mother*” and “*father*.” Martha said in her paragraph:

*I’ve drawn my mother and father because my family helps me feel strong, they make me feel very happy and I love them with my whole heart and I wish I was with them, the Bible also makes me happy I always pray that God helps me, I love reading the Bible and praying every day to gain strength*.

Clearly, the above paragraph shows that on a personal level, Martha *had faith* that enabled her to cope resiliently. She *read the Bible and prayed* to obtain strength. On the socioecological level, Martha remained resilient because she had *connections to her parents*, although she was not residing with them.

Mpee, a 17-year-old girl in grade 10 made a drawing of two human figures standing side by side. Mpee wrote the following paragraph:

*I have drawn a picture of my two caregivers, they really inspire me with many things, I would like to thank Mr XXX* [name withheld] *for the role of a father to me, thank you Daddy I really appreciate it and it means a lot to me, I’d also like to thank Miss XXX* [name withheld] *in many ways because when I am sad she is the one person who is there for me when I feel like the world has turned against me, she would tell me that she is there for me so thank you mommy I love you a lot and I will always do.*

On a personal level Mpee remained resilient because she *loved her caregivers* and had an *attitude of gratefulness*. The paragraph above shows that Mpee, on a socioecological level, had an *active support system comprising caregivers*.

**Figure 8:**
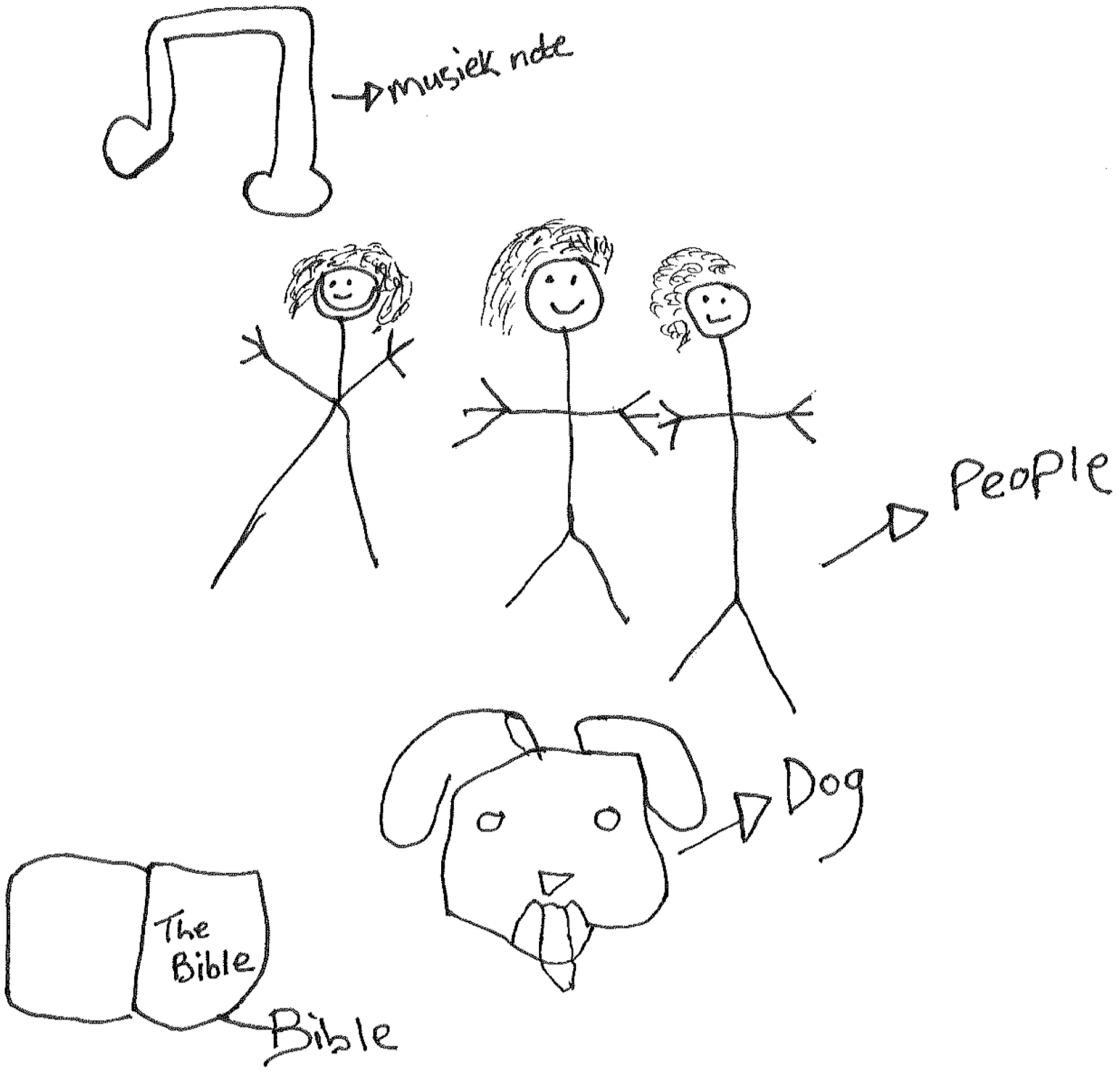
Drawing by Peaches.

Peaches, an 18-year-old boy in grade 12, made a drawing of three human figures and labeled them, “*people*,” the head of a dog, labeled “*dog*” and a book labeled, “*Bible*.” At the top of the drawing, there is a musical note, labeled, “*musiek note*,” meaning musical notes in English (Figure 8).

Peaches then wrote the following paragraph:

*Music is something beautiful, music makes me forget and gives me motivation and strength it relaxes and calms me, my friends and family support me they give me strength to keep fighting they always stand up for me, my dog’s barking makes me smile and gives me joy with that joy I can face another day. The bible, most of all I pray to God from straight above I talk to him and worship him, that how I fight through the hard days*.

On a personal level, *listening to music* enables him to forget his pain and be motivated, relaxed, and calm. *Religiosity* plays a role in enabling his resilience since he *prays, worships God*, and overcomes hardship. Peaches draws strength from his *friends and family*. It is interesting to note that his *dog has been integrated into his social ecology* and its barking gives the participant joy to face another day.

**Figure 9:**
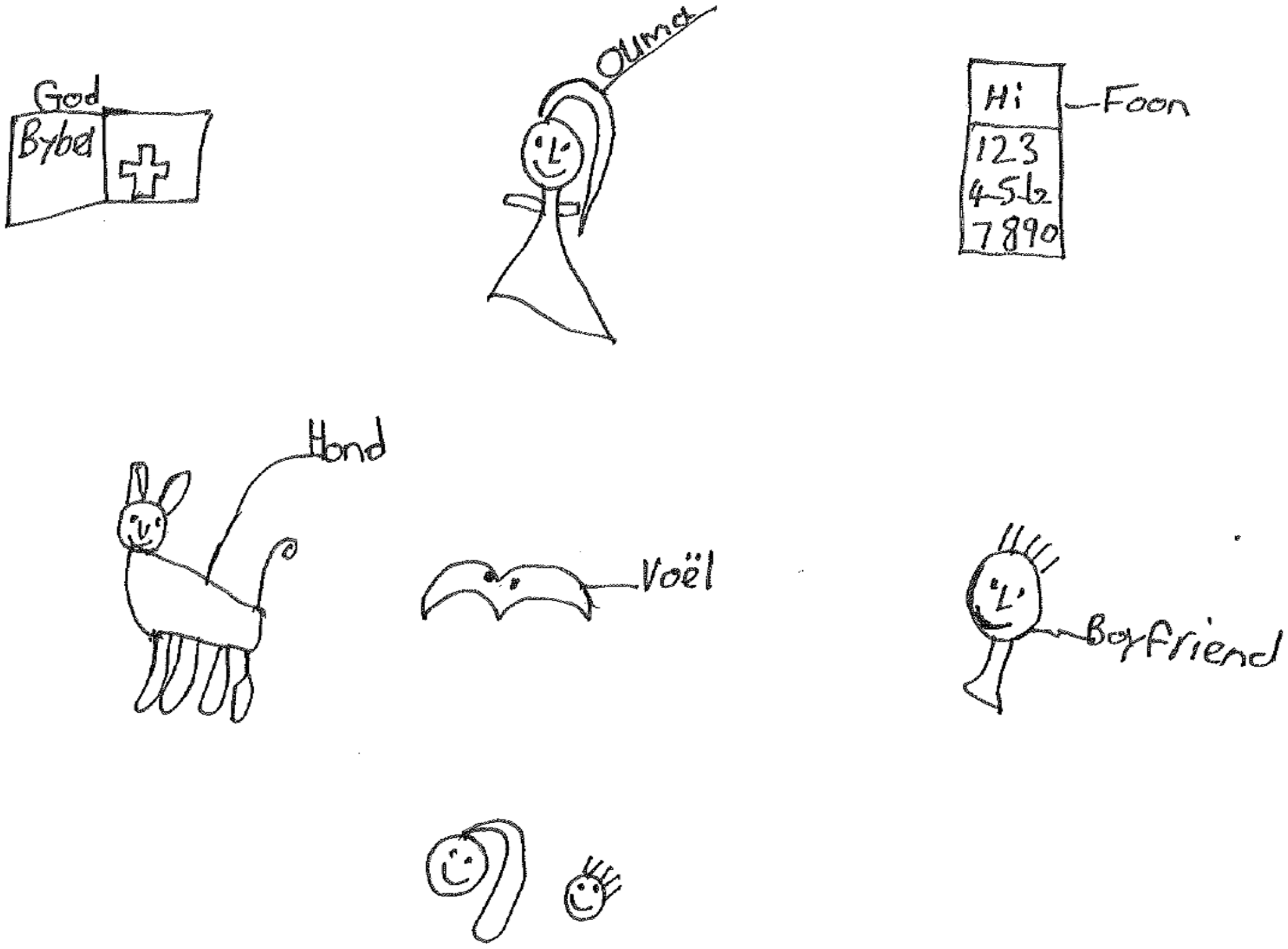
Drawing by Zahn.

Zahn, a 19-year-old girl in grade 12 made a drawing showing a book labeled “*God en Bybel*” [Bible in English], a female human figure labeled “*Ouma*” [grandmother], a mobile phone with numbers labeled, “*Foon”* [Phone], an animal labeled, “*Hond*” [Dog], a bird labeled “*Voël*” [Bird], a human figure labeled “*boyfriend*” and two human figure heads (Figure 9).

Zahn wrote the following paragraph:

*My God, want ek kan met hom praat en hy lig my op en hy antwoord mense se gebed* [My God, because I can talk to Him, and He lifts me up and He answers people’s prayers]. *My ouma, sy leer my hoe om vrou te wees. Sy moedig my aan* [My grandmother. She teaches me how to become a woman. She encourages me]. *My diere is my vriende en familie hulle liefde vir my.* [My animals are my friends and family, their love for me]. *Ook, my kêrel, hy ondersteun my* [My boyfriend too, he stands by me]. *My foon en musiek and kontak met my vriende* [My phone and music and contact with my friends]. *My gesin veral my sussie help my om sterk te staan* [My family, especially my sister; she helps me to stand strong].

On a personal level, Zahn coped by *listening to music* on her mobile phone. On the socioecological level, the participant had the benefit of social capital sourced from her *family, sister, friends, and boyfriend*. She *enjoyed her animals* too and considers them as friends.

Maugdoza, an 18-year-old boy in grade 11 made a drawing of gym equipment. Maugdoza then said below:

*Ek hou van gym, om gesond te wees, om slegte goed te vergeet, en vir my elke dag is n nuwe dag* [I enjoy the gym, to be healthy, to forget negative things and for me every day is a new day].

The statement above shows that on a personal level, Maugdoza coped by spending *time in the gym* to divert his thoughts from negative events and maintain health. He sees every day as new day. Researchers such as Lancaster and Callaghan (2022, p. 1) discovered that physical exercise promoted resilience during the COVID-19 pandemic.

**Figure 10:**
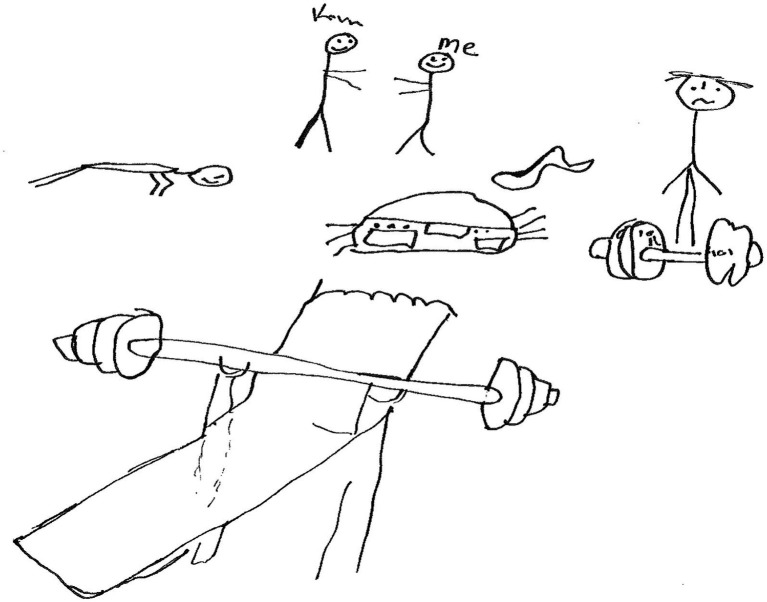
Drawing by the Rock.

The Rock, a 19-year-old boy in grade 11, made a drawing with 4 human figures, a radio and gym equipment too (Figure 10).

The Rock then wrote the following:

*Wat my help om te cope en deur my gevoelens en myself te werk, is musiek* [What helps me to cope and work through my emotions is music]. *Dit maak my lewe bietjie maklik net deur oefen in die gym en musiek luister* [What makes my life easy is working out in the gym and listening to music].

The Rock coped on a personal level through *working out in the gym* and *listening to music*. As he pointed, the gym and working out made his life easier.

Tumi, a 17-year-old boy made a drawing of a face mask. He wrote “*No mask*; *no entry*” on it. Tumi then wrote the following statement:

*I drew a mask because it helped me not to spread the virus to other people.*

The statement shows that Tumi relied on *his face mask* to avoid infection and spreading the COVID-19 virus.

**Figure 11:**
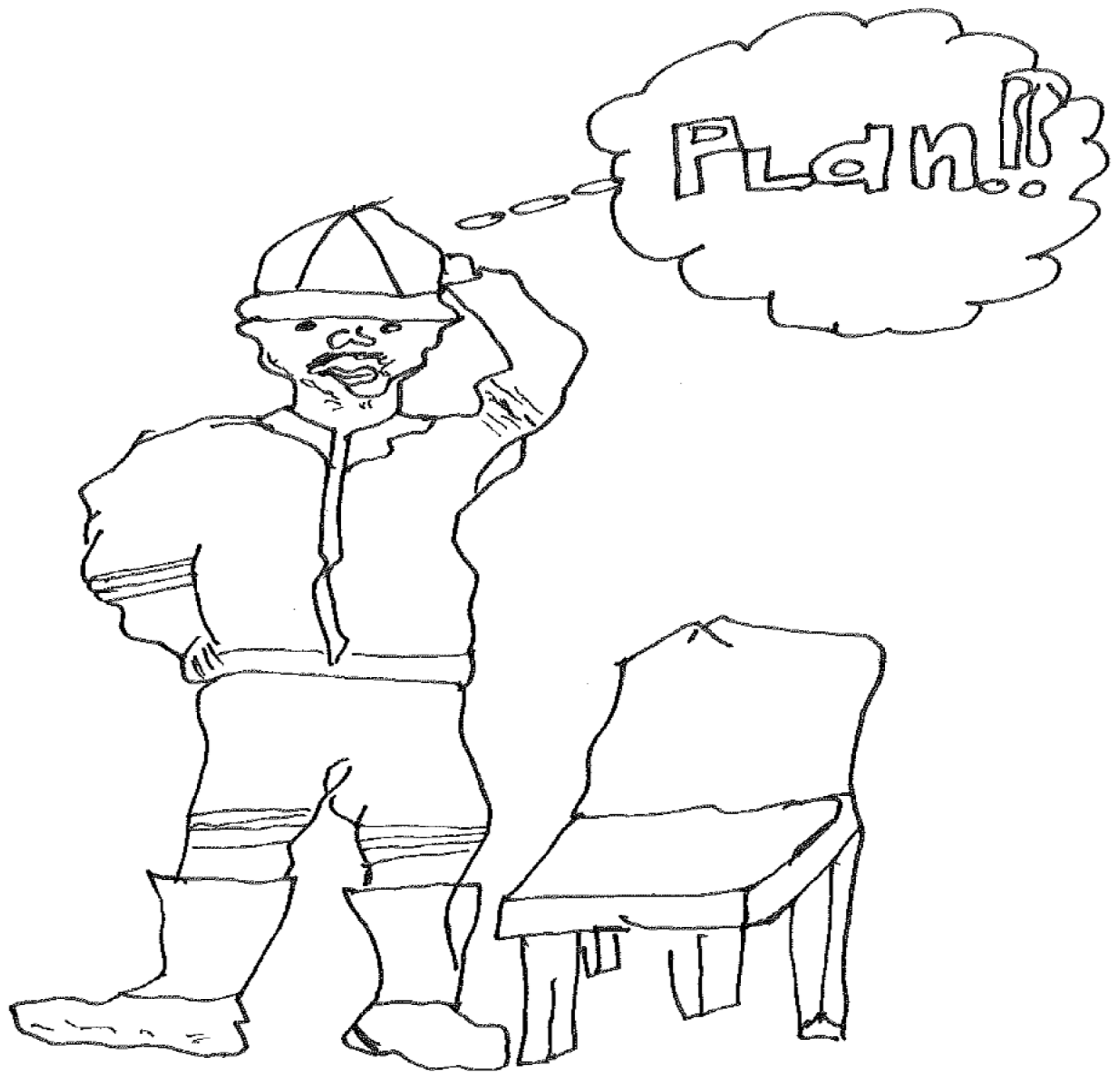
Drawing by Hloni.

Hloni, a 16-year-old boy in grade 11 made a drawing of a male human figure standing next to a chair. A bubble above its head has the word, “Plan!!” inside (Figure 11).

Hloni wrote the following:

*The person in my drawing is my uncle he is the one who makes me happy and is always by my side, he always told me to make a plan whenever I’m in a difficult situation*.

The above statement shows that Hloni remained resilient in the context of risk because his *uncle was always there to support* him. His uncle advised him to *“make a plan”* when he faced a difficult situation.

**Figure 12:**
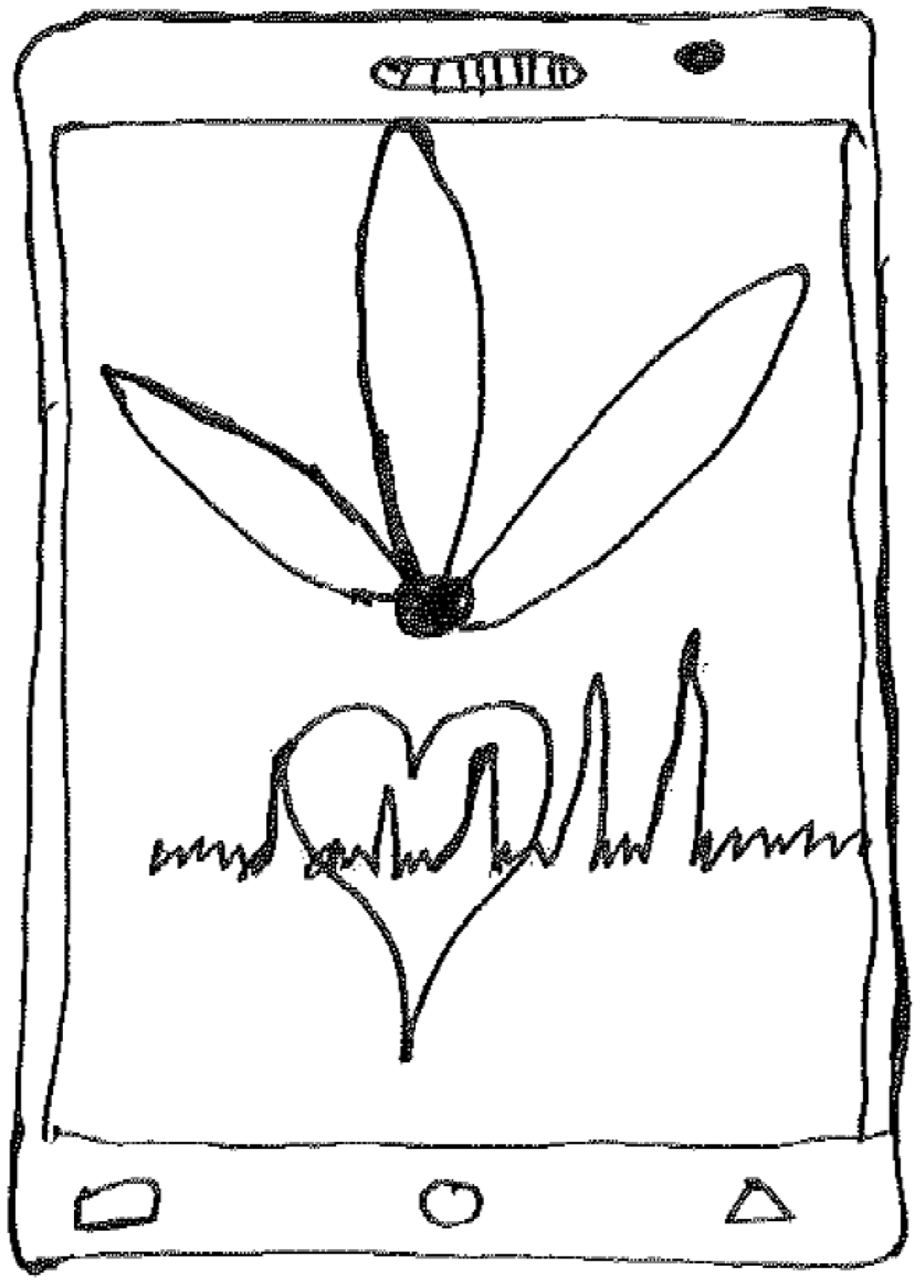
Drawing by Jagghlik.

Jagghlik, an 18-year-old boy made a drawing of a mobile phone (Figure 12).

Jagghlik wrote the following:

*Wat my laat by hou in die lewe is wanneer ek meer met my foon speel sodat ek net kan vergeet wat in my lewe aangaan en net meer probleme en stress vir minder en nie onder depressive lei nie* [What helps me to hold on in life, is when I play with my mobile phone, so that I can forget what is going on in my life, and just more problems and stress to decrease so that it does not lead to depression].

Jagghlik used his *mobile phone* as a diversion and a tool to connect to others and cope on a personal level. This he did to reduce problems and stress, to avoid the onset of depression.

**Figure 13:**
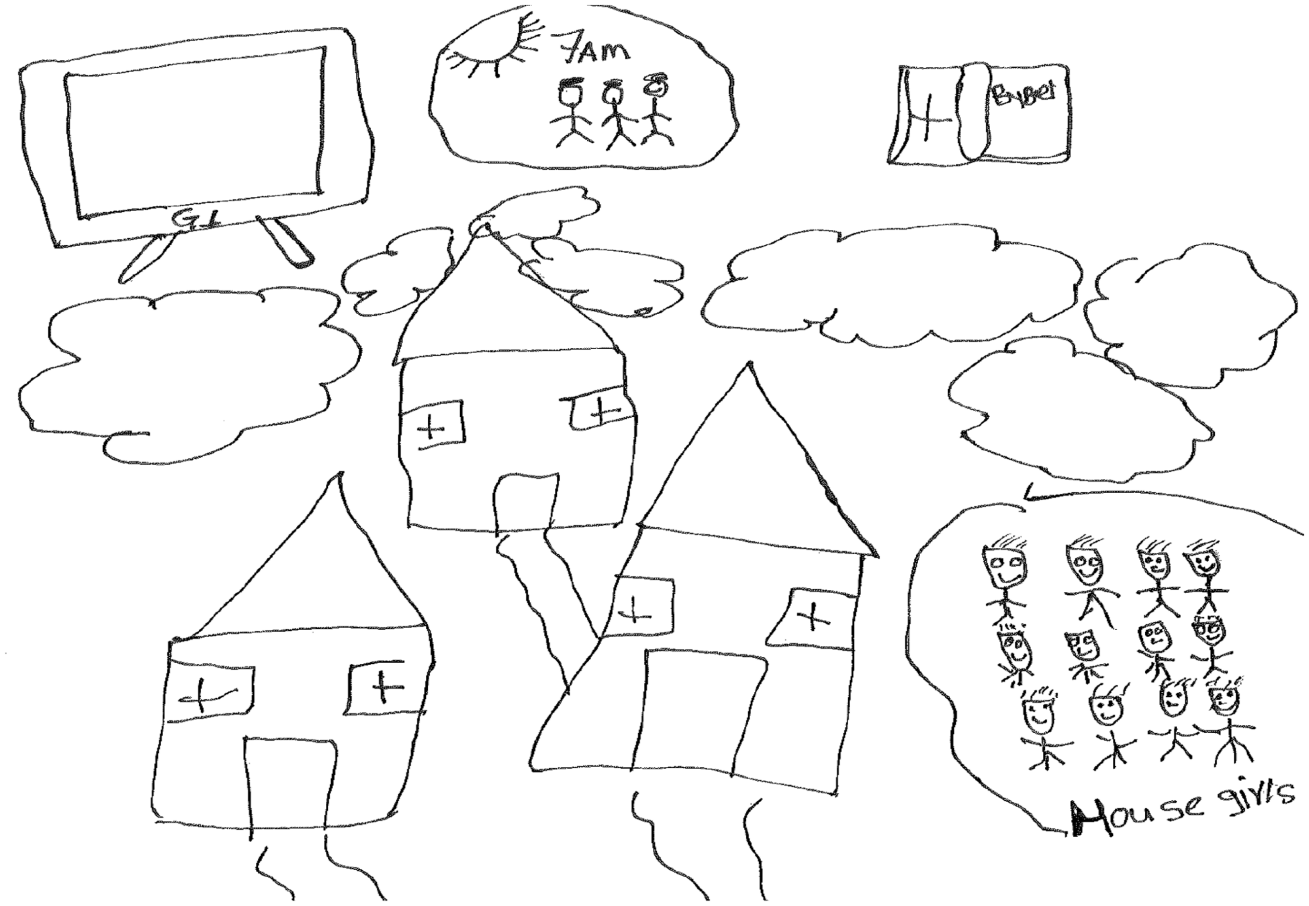
Drawing by Tony.

Tony, a 17-year-old boy made drawings of a television set, three houses, clouds, the sun, human figures and a Bible (Figure 13).

Tony wrote the following paragraph:

*Deur TV te kyk, deur my familie by my te hê, die Bybel help my* [By watching tv (television), having my family with me, the Bible helps me]. *Want ek het die Here se ondersteuning* [Because the Lord supports me], *ek wens altyd om in ŉ nuwe huis te bly en om saammet die kinders, en my vriende in diselfde huis te bly* [I wish to live in a big house and together with other children, and friends in the same house].

On a personal level, Tony coped by *watching television* and *reading the Bible*. He believed the Lord supported him. It does seem as if he wished to live in a big house with other children and friends. Sigre-Leirós et al. (2022, p. 10) noted that during the COVID-19 pandemic watching television served to mitigate the impact of the pandemic-induced isolation however, binge-television watching was less effective in enhancing adaptive coping.

**Figure 14:**
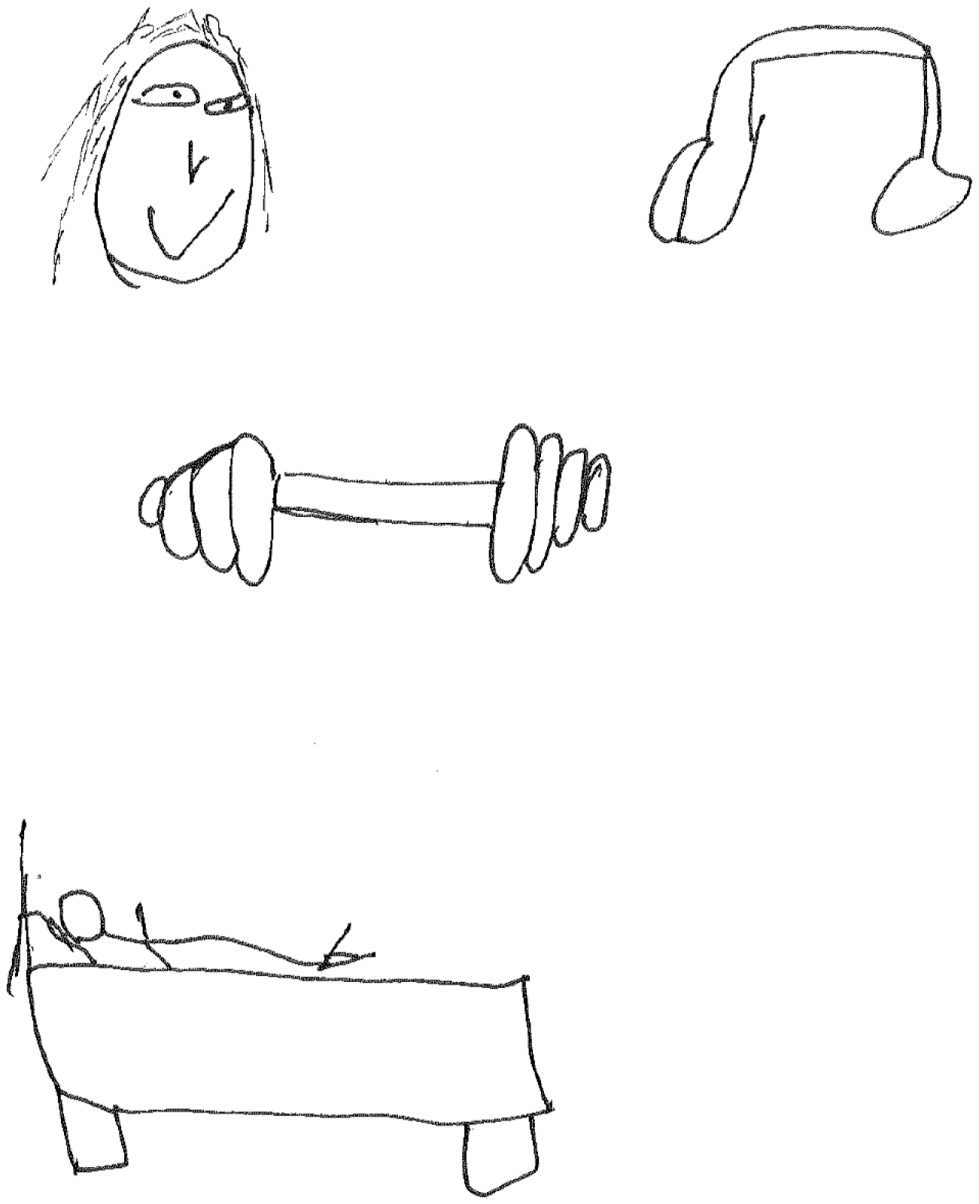
Drawing by Pottas.

Pottas, 17-year-old boy made a drawing involving a human head, headphones, gym equipment and a human figure lying on a bed (Figure 14).

Pottas said the following:

*When I gym it helps me to get rid of all my anger, listening to music helps me to keep my focus, a friend of mine is always listening when I need it the most and sleeping also helps me to not put my aggression onto other people if I cannot do any of the above-mentioned activities.*

It is evident that Pottas coped with anger through *working out in the gym* and *listening to music* to help him focus. He dealt with aggression by *sleeping*.

**Figure 15:**
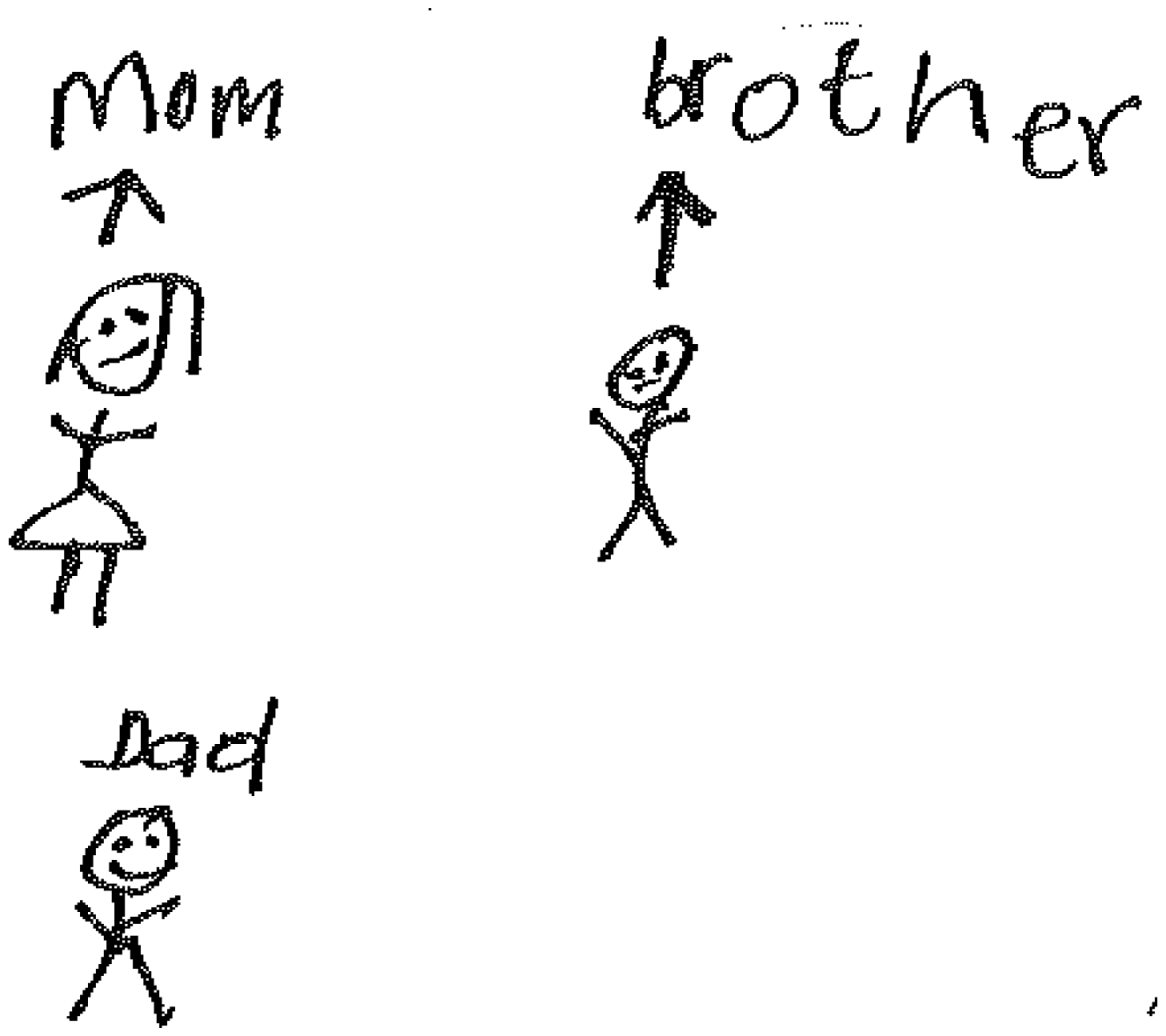
Drawing by Zwile.

Zwile, a 14-year-old girl in grade 8, made a drawing of three human figures. The human figures are labeled “*Mom*,” “*brother*,” and “*dad*” (Figure 15).

Zwile wrote the following words on her drawing:

*What makes me feel better are my mom, brother and dad. It makes me feel better by giving me a hug, and wish me good night, prays for me at night, feeds me and take care of my body.*

On a socioecological level, Zwile coped through *social support from her brother and parents*. The parents gave her a hug and prayed for her whenever they were around. They tried their best to care for the participant.

## Discussion of findings

The purpose of our exploratory study was to document anchors of resilience in children residing in an out-of-home care institution. We were interested in determining factors and processes that enabled them to develop resilience and remain resilient seeing that they had been affected by the COVID-19 pandemic that disrupted development in young people worldwide. The main finding is that the participants resiliently coped with their lives due to complex combinations of personal strengths and socioecological resilience resources or anchors.

Table 1 summarizes the personal and socioecological resources or anchors that enabled the resilience of the participants.

**Table 1:** Personal and socioecological resilience resources.

Personal resilience resources/anchors	Socioecological resilience resources/anchors
An attitude of gratefulness	Having positive relationships
Comfort eating of sweets	Loving hugs from parents
Concentrating on studies	Parents praying for them
Desire to succeed	Perceived connectedness to parents
Face mask	Seeing pets as friends
Future focus	Support from peers
Having a safe place	Support from sister
Having religious faith	Support from uncle
Hope for a better life	Support from caretakers
Lessons from stories read	Having bonds with pets
Listening to music	
Love for animal or pets	
Love for people	
Making a plan	
Not focusing on negatives	
Optimism	
Perceived care	
Prayerfulness	
Reading motivational quotations	
Reading stories	
Reading the Bible	
Seeing every day as a new day	
Singing	
Sleeping	
Social media	
Watching television	
Working out in the gym	
Writing songs	

From Table 1 a number of themes or resilience anchors can be inferred. For instance, in terms of personal anchors, it is evident that religion—as part of culture—played a prominent role in enabling resilience in the participants. Four participants mentioned prayer, worshipping, reading the bible and listening to gospel music as anchors for their resilience. Having faith has been linked to resilience in other South African studies involving children in care institutions (Malindi and Theron, 2010, p. 323). Other studies that documented religious faith include the study by Gunnestad and Thwala (2011, p. 169) involving young people in Zambia and Eswatini. It is important to note that these studies occurred prior to the COVID-19 pandemic, except one by Ilashenko et al. (2021, p. 5). The study by Ilashenko et al. (2021, p. 5) made a similar finding in a study that occurred during the COVID-19 pandemic and added that religious practices enabled participants to calm down, and thus cope resiliently amidst the fear and uncertainty that the pandemic produced.

A second strong personal anchor nurtured by a number of participants was to cope via music—either listening to music, singing or writing songs. In this regard, a participant specifically mentioned that listening to gospel songs brought back her joy. It was not clear how music was listened to—but some participants mentioned using their cellphones for this. Headphones were sometimes used too. This anchor enabled a number of participants to cope and develop their capacities to resile—albeit through escaping reality for periods of time. Music has been reported in other studies (Kegelaers et al., 2021, p. 1,279; Rosenberg et al., 2021, p. 6) that did not necessarily sample youth in care institutions. Of these two studies, it is the study by Rosenberg et al. (2021, p. 6) that reports that music enabled resilience and this shows that music is an accessible anchor of resilience.

A third common personal (and perhaps socioecological) anchor was to spend time on one’s cellphone and engage in relationships via social media. Various social media platforms were utilized with Facebook being the most popularly used to connect with family members. Previous studies have shown how people can resile in the context of disaster through virtual networks (Dufty, 2012, p. 40; Jurgens and Helsloot, 2017, p. 79; Mano, 2020, p. 460; Senekal et al., 2022, p. 10–11). It can be argued that through virtual networks people can maintain meaningful connections that enable adaptive coping.

Some participants related that they tried to focus on certain activities to cope. For instance, a workout in the gymnasium was mentioned by two participants. In our study, working out was used to relieve anger, in like manner, Lancaster and Callaghan (2022, p. 1) note that physical exercise enabled resilience in the context of the COVID-19 pandemic. It does seem as if working out can be protective in different contexts.

Sleeping was mentioned by more than one participant as a way to cope adaptively. While other studies show that sleeping enables adaptive coping (Guida et al., 2023, p. 3), sleeping in our study was used as an escape mechanism. Another escape mechanism that our participants used to cope resiliently was comfort eating. It appears as if comfort eating is a response to stress. For example, there is a previous study that notes the protective function of comfort eating in the context of the COVID-19 pandemic (Salazar-Fernández et al., 2021, p. 1).

A few participants mentioned that watching television enabled them to remain resilient. In a preceding study by Sigre-Leirós et al. (2022, p. 10) the findings show that watching television had two sides to it. On the one hand it enabled coping but on the other it rendered at risk people more vulnerable especially if it is overdone.

Reading of especially stories, and motivational quotations was mentioned as a way in which other participants coped resiliently. Reading can inspire one who is at risk and provide useful information on adaptive coping resources (Tovey, 2021, p. 8).

The strongest and most common socioecological anchor expressed by most participants was to connect with their caregivers, family and friends in one way or the other—despite having minimal time in their physical presence. This happened mostly via cellphone communication, but personal touch and hugs were also mentioned when family members were able to meet in person. Interestingly it was not always direct family that was mentioned, but a significant extended family member such as an uncle with whom a very strong bond existed.

A number of participants consciously focused on constructive intrapersonal attitudes, such as gratefulness, a desire to succeed, focusing on the future, clinging to hope for a better life in future, not focusing on negatives, making a plan and choosing to be optimistic. Kumar et al. (2022) found that gratefulness served as a resilience anchor during COVID-19. Our findings therefore corroborate this finding although our study involved youth in care.

Historically, the main aim of a child welfare system has been to enhance the wellbeing and safety of children and youth at risk (Leve et al., 2012, p. 1,208). In some instances, however, there have been reports of young people being maltreated in these institutions (Gusler et al., 2020, p. 455). In contrast to the latter, the out-of-home group home care institution where the participants resided seemingly served as a microsystemic stronghold (Theron and Engelbrecht, 2012, p. 265) that provided opportunities for social support. In our study, social capital was sourced from a boyfriend, girlfriend, sister, brother, an uncle, friends, mothers, fathers, grandmother, and caregivers. A similar finding was made in South African studies by Moodley et al. (2020, p. 47) and van Breda and Theron (2018, p. 1).

Following an extensive review of South African studies, van Breda and Theron (2018, p. 1), demonstrated the intersectionality of personal, relational, structural, and spiritual or cultural enablers of resilience in young people, although the studies reviewed preceded the COVID-19 pandemic. This is further evidence that, in line with the SERT’s principle of decentrality (Ungar, 2011, p. 4), the capacity to cope resiliently depends not only in what is built inside children but also in what is built around them. Therefore, it should be accepted that children tend to flourish in the context of risk when they have strong social support systems and access to multiple services in their localities (Theron et al., 2022, p. 7).

Having strong relationships with animals was also mentioned as a socioecological (and perhaps personal) resilience anchor. It is interesting to note that our finding was made in earlier studies (Thompson et al., 2014, p. 215; Applebaum et al., 2021, p. 2) involving participants in their original homes.

Typically, care institutions do not have enough resources; and the fact that the care institution where the participants resided enabled the participants to develop resilience is evidence of the complexity of the resilience phenomenon and the processes (i.e., risks and promotive/protective factors) linked to it according to the SERT (Ungar, 2011, p. 1). Little was made by participants of the risks they faced, however, they referred to choosing loneliness, sadness, anger and aggression and ways in which they coped with these risks.

In contexts where options are minimal, some young people resort to coping with risk in ways that could be seen as atypical (Malindi and Theron, 2010, p. 324; Ungar, 2011, p. 7–8). In this study, the young people did not report many mechanisms that could be seen as atypical according to the SERT (Ungar, 2011, p. 7–8), however, they mentioned escaping reality, comfort eating and choosing loneliness, that in this context, could be seen to be atypical.

It is important to note that the participants in our study were all still minors, and that the care institution provided them with the protection and care they needed to resile in the context of risk. However, it was not clear whether they were exposed to managed opportunities for independent living beyond care (Hlungwani and van Breda 2022, p. 137). A South African study by Hlungwani and van Breda (2022, p. 137) found that involving youth in care in programs that prepare them life beyond care enabled resilience. It prepares them to be productive in the open society with all the changes in it.

Finally, while our study reports promotive factors that are well-known and universal in some instances, it should be noted that these factors were based on studies involving young people who were either not affected by the COVID-19 pandemic or not in care institutions. Furthermore, the findings wise us that the resilience of young people can be meaningfully sustained in future disasters if access to combinations of personal and socioecological is enabled.

### Limitations

Although this study makes interesting findings, it was not without limitations. The study was conducted in a group home care institution that is well resourced in terms of professionals and finances. The findings should therefore be seen within this context. The findings may be transferable to similar institutions, but care should be taken when trying to transfer them to care institutions that have less access to resources and thus fall short of their mandate. The method used to co-generate data was least intrusive and child-friendly, however, findings from studies that use a single method to generate data ask for replication and the use of multiple methods to generate data.

## Conclusion

The findings of this study suggest that youth in an out-of-home care group home setting developed and remained resilient in the context of risk due to certain personal and socioecological resources/anchors. The findings do not point to evidence that they maintained resilience via other role players outside of the care institution. For example, they did not mention how their schools enabled them to cope and how the care institution connected them to community services and resources except the school. It is not clear from these research findings whether these role players and services were obvious to them—or whether their personal and care setting-related anchors were viewed as adequate.

The findings suggest that the participants’ coping strategies were often gender specific. Boys listened to music and went to the gym more than girls. Girls listened to music too, but they chose to focus strongly on their mobile phones. It was interesting to note that some participants coped through strong focuses on a grateful attitude and having bonds with animals.

Of particular interest was the fact that one participant thanked the primary researcher for the privilege to take part in the research. This may suggest that the draw-and-write technique enabled the participants to perform a cognitive appraisal of their situations, voice their opinions and achieved a measure of catharsis.

Finally, we recognize the need for the study to be replicated with more varied child-friendly methods, in more diverse care settings and with a focus on the role of multisystemic interventions and services.

## Data availability statement

The original contributions presented in the study are included in the article/supplementary material, further inquiries can be directed to the corresponding author.

## Ethics statement

The studies involving human participants were reviewed and approved by Community-based Educational Research at North-West University. Written informed consent to participate in this study was provided by the participants’ legal guardian/next of kin.

## Author contributions

All authors listed have made a substantial, direct, and intellectual contribution to the work and approved it for publication.
